# Sandfly-Borne Phlebovirus Isolations from Turkey: New Insight into the *Sandfly fever Sicilian* and *Sandfly fever Naples* Species

**DOI:** 10.1371/journal.pntd.0004519

**Published:** 2016-03-23

**Authors:** Cigdem Alkan, Ozge Erisoz Kasap, Bulent Alten, Xavier de Lamballerie, Rémi N. Charrel

**Affiliations:** 1 UMR "Emergence des Pathologies Virales" (EPV: Aix-Marseille University—IRD 190—Inserm 1207—EHESP), Marseille, France; 2 Fondation IHU Méditerranée Infection, APHM Public Hospitals of Marseille 13385, Marseille, France; 3 Faculty of Science, Department of Biology, Ecology Section, ESR Laboratories, Hacettepe University, Ankara, Turkey; Institut Pasteur, FRANCE

## Abstract

Southern Anatolia in Turkey at the border with Syria, where many refugee camps are settled, is endemic for sandfly-borne leishmaniasis. Sandfly-borne phleboviruses are also known to circulate in this region, although their relevance in terms of medical implications is virtually unknown. Therefore, the specific objectives of our study were firstly to identify isolate and characterise potentially pathogenic phleboviruses in sandflies; secondly to determine the complete genomic sequence of any viruses that we were able to isolate; and thirdly, to further our understanding of the potential medical importance and epidemiological significance of these viruses. To achieve these objectives, we organised field campaigns in 2012 and 2013. Two new phleboviruses (Toros and Zerdali viruses) were isolated and characterized by complete genome sequencing and phylogenetic analyses. Toros virus was genetically most closely related to Corfou virus within the Sandfly fever Sicilian group. Zerdali virus was most closely related to Tehran virus within the *Sandfly fever Naples* species. Although these new viruses belong to genetic groups that include several human pathogens, it is not yet clear if Toros and Zerdali viruses can infect humans and cause disease such as sandfly fever. Consequently, the availability of these genetically characterized infectious viruses will enable seroprevalence studies to establish their medical importance in this region and to assist the health agencies to develop appropriate and effective disease control strategies.

**Background:**

Many studies have presented virus sequences which suggest the existence of a variety of putative new phleboviruses transmitted by sandflies in the Old World. However, in most of these studies, only partial sequences in the polymerase or the nucleoprotein genes were characterised. Therefore to further our understand of the presence and potential medical importance of sandfly-borne phleboviruses that circulate in southern Anatolia, we initiated field campaigns in 2012 and 2013 designed to identify, isolate and characterise phleboviruses in sandflies in this region

**Methodology/Principal Findings:**

An entomological investigation encompassing 8 villages in Adana, Mediterranean Turkey was performed in August and September 2012 and 2013. A total of 11,302 sandflies were collected and grouped into 797 pools which were tested for the presence of phleboviruses using specific primers for RT-PCR analysis and also cell culture methods for virus isolation. Seven pools were PCR positive, and viruses were isolated from three pools of sandflies, resulting in the identification of two new viruses that we named Zerdali virus and Toros virus. Phylogenetic analysis based on full-length genomic sequence showed that Zerdali virus was most closely related with Tehran virus (and belongs to the *Sandfly fever Naples* species), whereas Toros virus was closest to Corfou virus.

**Conclusions/Significance:**

The results indicate that a variety of phleboviruses are co-circulating in this region of southern Anatolia. Based on our studies, these new viruses clearly belong to genetic groups that include several human pathogens. However, whether or not Toros and Zerdali viruses can infect humans and cause diseases such as sandfly fever remains to be investigated.

## Author Summary

We provide evidence that sandfly-borne phleboviruses belonging to 3 distinct genetic and phylogenetic groups (Sandfly fever Naples virus [SFNV], Sandfly fever Sicilian virus [SFSV], and Salehabad virus [SALV]) co-circulate in Adana city, in Mediterranean Turkey. While Adana virus was recently described as a new member of the SALV species, Zerdali and Toros viruses are described here as new phleboviruses genetically closely related to SFNV and SFSV, respectively. In this study, isolated and characterised these two new viruses by determining their complete genome sequence and by phylogenetic analysis. This study demonstrates that 3 distinct viruses can co-circulate in the same geographic area and based on their phylogenetic relationships and association with sandflies are likely to be transmitted by these arthropod vectors. Our molecular and phylogenetic data are important for establishing group-specific molecular detection assays in order to further understand of the possible impact of these viruses in animal and human health in this region of Turkey.

## Introduction

The genus *Phlebovirus* (family *Bunyaviridae)* currently contains 9 viral species *Sandfly fever Naples* (SFNV), *Salehabad* (SALV), *Rift Valley fever* (RVFV), *Uukuniemi* (UUKV), *Bujaru* (BUJV), *Candiru* (CRUV), *Chilibre* (CHIV), *Frijoles* (FRIV) *and Punta Toro* (PTV) including 33 distinct serotypes, and 32 tentative serotypes as defined in the 9th Report of the International Committee on Taxonomy of Viruses (ICTV) [1]. Nevertheless, the past decade has witnessed the discovery of many new phleboviruses that remain to be classified: some are transmitted to vertebrates by sandflies (Fermo (FERV), Granada (GRAV), Punique (PUNV)) [2, 3, 4], others by ticks (Heartland (HRTV), Hunter island group (HIGV)) [5, 6], whereas some do not have recognised vectors and appear to be transmitted directly between vertebrates (Malsoor (MALV), Salanga (SGAV)) [7, 8].

In the Old World, sandfly-borne phleboviruses are transmitted between vertebrates mainly by female sandflies (genus *Phlebotomus*) when they take a blood meal. Some Old World sandfly-borne phleboviruses may cause self-limiting febrile illnesses (sandfly fever) or neuro-invasive infections. They are widely distributed in the Mediterranean Basin, in Africa, in the Indian subcontinent, in the Middle-East, and in far-eastern former USSR republics [9]. Annually, Toscana virus (TOSV), a serotype of SFNV is the leading cause of meningitis from May to October in central Italy [10] and one of the most prevalent human pathogenic phleboviruses in other southern European countries.

Forty years ago, seroprevalence studies showed that Sandfly fever Sicilian virus (SFSV) and SFNV were present in the Mediterranean and Aegean regions of Turkey [11, 12]. Recently, serological investigations were carried out in the Mediterranean, Aegean, and Central Anatolian regions, where outbreaks have occurred and circulation of SFSV and a SFS-like virus (Sandfly Fever Turkey virus (SFTV)) were reported [13,14,15]. The presence of TOSV was confirmed serologically and through RNA detection and sequencing [16,17,18,19]. Despite the publication of many articles, virus isolations were reported only for SFTV from a patient [13] and Adana virus (ADAV) [20] from sandflies. To further understand of the dynamics of sandfly-borne phleboviruses and sandfly fever in the Mediterranean region in the vicinity of Adana city, we organized sandfly trapping campaigns.

## Materials and Methods

### Sandfly trapping

**Fig 1:**
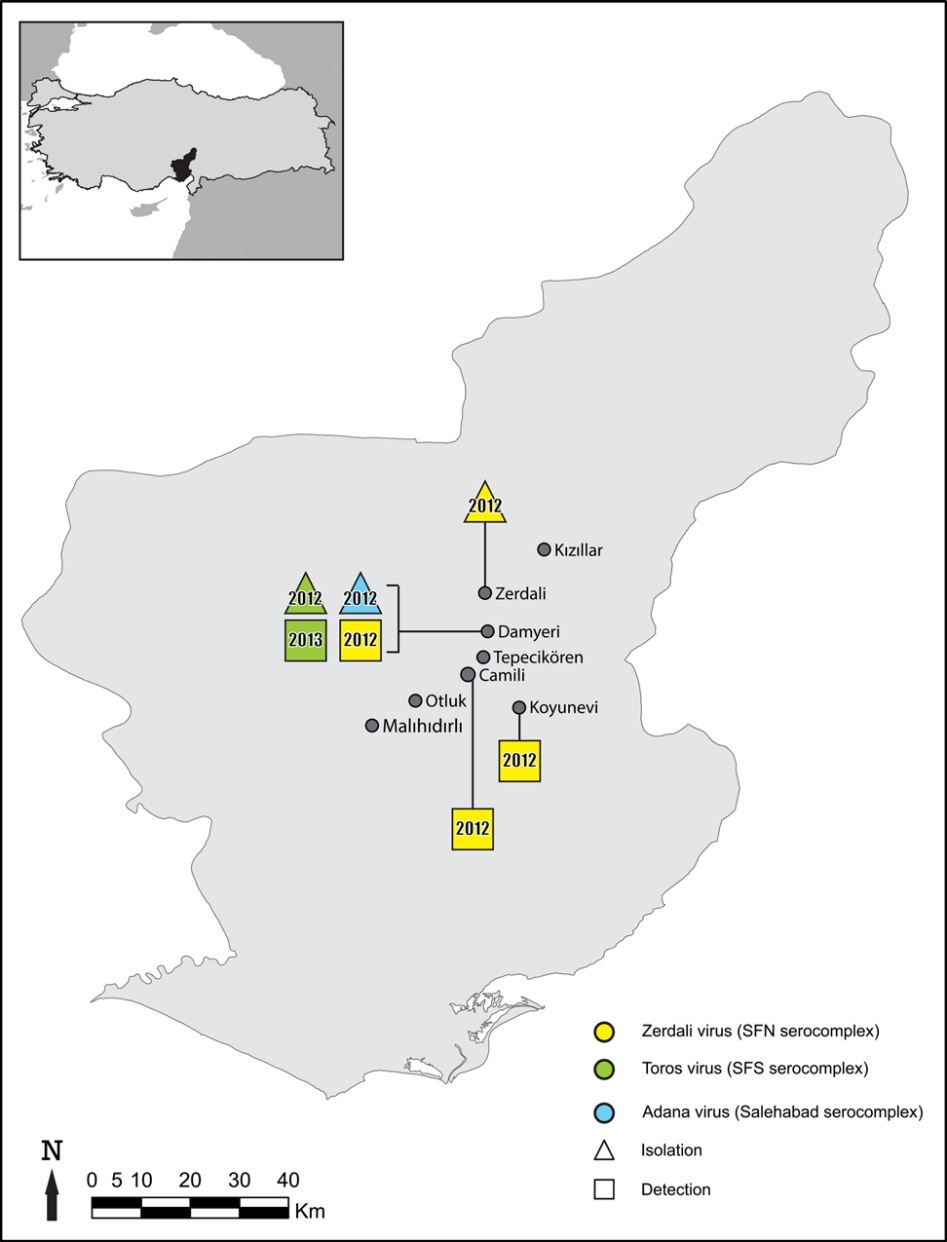
Geographic representation of the results.

Sandflies were captured during August and September in 2012 and in 2013 in Adana city located in the Mediterranean region of Turkey (Fig 1) using CDC Miniature Light Traps as previously reported [21]. Live sandflies were pooled according to sex, trapping site and day of capture, with up to 30 individuals per pool and placed in 1.5mL tubes, and stored at -80°C. In order to reduce the time between capture and storage and therefore to increase the likelihood of virus isolation, morphological identification of the sandflies was not performed.

### Genetic detection of phleboviruses

Sandfly pools were processed as previously described [22] in a final volume of 600μL, of which 200μL were used for total nucleic acid extraction using the Virus Extraction Mini Kit the BioRobot EZ1-XL Advanced (both from Qiagen). Elution was performed in 90μL of extraction buffer of which 5μL were used for RT-PCR and nested-PCR assays using primers targeting the polymerase gene and the nucleoprotein gene as previously described [20, 23, 24]. PCR products of the expected size were column-purified (Amicon Ultra Centrifugal filters, Millipore) and directly sequenced.

### Specific detection of new phleboviruses

Two real-time RT-PCR assays (Rt-RT-PCR) were designed for specific detection of the new strains in the N gene: TORV-N-FW (AACTCTGACTCGTGTGGCTG), TORV-N-REV (GCCTTGGGTATGTCTGACCA), and TORV-N-Probe (6FAM-AGGCAATAGAAGTTGTGGAGAAC-TAMRA); ZERV-N-FW (ACTTCCTGTTACTGGAACAACAAT), ZERV-N-REV (CCATGAGCATCTGCAATAACTTC), and ZERV-N-Probe (6FAM-ATGATGCATCCTAGTTTTGCAGGA-TAMRA). Reaction conditions and cycling programs were previously described [20].

### Virus isolation

Fifty μL (derived from sandflies ground in the 600μL of EMEM as aforementioned) were inoculated onto a 12.5 cm^2^-flask of Vero cells, incubated at room temperature for 1 hr, and supplemented with 3mL of EMEM (5% FBS, 1% Penicillin/Streptomycin, 1% L-Glutamine 200 mM, 1% Kanamycin, and 3% Fungizone). The flasks were incubated at 37°C in 5% CO_2_ atmosphere and examined daily for cytopathic effects (CPE).

### Complete genome sequencing

For detailed characterisation, Zerdali virus (ZERV) passage 5, Toros virus (TORV) strain 292, passage 3, and TORV strain 213, passage 7 were subjected to complete genome characterisation using Next Generation Sequencing (NGS). Briefly, 140μL of infectious cell culture supernatant medium was incubated with 30 U of Benzonase (Novagen 70664–3) for 7 hr at 37°C.This material was then purified using the Viral RNA Mini Kit (Qiagen). Tagged random primers for reverse transcription (RT) and tag-specific and random-primers were used for PCR (Applied Biosystems). The resulting PCR products were purified (Amicon Ultra Centrifugal filters, Millipore); 200ng of DNA were used for sequencing using the Ion PGM Sequencer (Life Technologies SAS, Saint Aubin, France). NGS reads, of 30 nucleotides minimum length, were trimmed using CLC Genomic Workbench 6.5, with a minimum of 99% quality per base and mapped to reference sequences. Parameters were set such that each accepted read had to map to the reference sequence for at least 50% of its length, with a minimum of 80% identity to the reference. From the contigs obtained, viral sequences were identified by best BLAST similarity against reference databases. Sequence gaps were completed by amplification and sequencing overlapping regions using either Sanger sequencing or NGS. The 5' and 3' extremities of each segment were sequenced using a primer including the 8-nt conserved sequence as previously described [25].

Complete genome sequencing was also performed for Corfou virus (CFUV) strain PaAr814 using the frozen cell culture supernatant medium following the methods above for comparison with the newly discovered TORV strains since they were shown to be closely related but only the complete S [26], the partial L [4] and M [27] genome sequences of the CFUV were known. Ultimately, all complete sequences obtained using NGS were verified by amplification and Sanger sequencing of overlapping regions spanning the entire genome.

### Calculation of genetic distances and Phylogenetic Analyses

Complete sequences of each of the 5 genes (L, Gn, Gc, N, Ns) were aligned without indels together with homologous sequences of selected phleboviruses retrieved from the Genbank database using CLUSTAL within the MEGA 5 program [28]. Nucleotide (nt) and amino acid (AA) distances were calculated with the p-distance method. Neighbor-joining (p-distance model) and Maximum likelihood analyses were carried out with AA sequences using MEGA version 5, with 1000 bootstrap pseudoreplications.

### Recombination analysis

The Recombination Detection Program v.4.27 (RDP4) was used for recombination analysis using the nucleotide alignments. Recombination events, likely parental isolates of recombinants, and recombination break points were analyzed using RDP, GENECONV, Chimaera, MaxChi, BOOTSCAN, and SISCAN algorithms implemented in the RDP4 program with default settings [29].

### Genotyping of sandflies in the virus-positive pools

To attempt identification of the sandfly species present in the TORV and ZERV positive pools, PCR was performed using 3-μL of nucleic acid extract of the pool to amplify the cytochrome c oxidase I (COI) gene using the following primers; LCO1490: GGTCAACAAATCATAAAGATATTGG and HCO2198: TAAACTTCAGGGTGACCAAAAAATCA [30]. The PCR products were processed and sequenced through NGS as described above. NGS reads were compared with available sequences in Genbank by Blastn using the CLC Genomic Workbench 6.5. For the final determination of the species the sequences were aligned with the reference sequences of regional populations of the sandfly species.

However, we would like to acknowledge that a valid protocol would be to cut off the male genitalia using a cold-stage microscope in the laboratory, so that the specimens can be identified morphologically. This would be faster and cheaper than PCR amplification followed by NGS or Sanger sequencing of the COI gene.

## Results

### Sandfly trapping and virus detection

A total of 11,302 (4,513 females and 6,789 males) sandflies were collected in August and September 2012 and 2013 from eight villages (Fig 1) located in the surroundings of Adana city (Mediterranean Turkey). They were organized as 797 pools (494 females, 303 males) (Table 1). Two pools, #213 and #292, were positive with primers N-phlebo2S/2R and N-phlebo1S/1R [24], respectively. The 245-nt sequence obtained from pool #213 was most closely related with CFUV (Genbank no: GQ165521; 95% AA identity, 78% nt identity). The 513-nt sequence corresponding to pool #292 was also closely related with CFUV (Genbank no: GQ165521; 88% and 78% identity at the AA and nt level, respectively). These 2 pools consisted each of 20 male sandflies trapped in Damyeri village in 2012 (36S0733357 North and 4140570 East, altitude 194m).

The pool #37 (20 males trapped in Zerdali village in 2013; 36S732947 North and 4142749 East, altitude 238m) was positive using primers SFNV-S1/S2 [23]. The corresponding 390-nt sequence was most closely related to THEV (Genbank no: JF939848; 96% AA identity, 85% nt identity).

**Table 1:** Distribution of the sandfly specimens and pools according to the sampling locations in Adana, Mediterranean region of Turkey in 2012 and 2013.

Village	Number of collected sandflies			Number of pools		
**2012**	**Female**		**Male**	**Female**		**Male**
**Damyeri**	1,974		2,500	99		123
**Zerdali**	697		692	35		34
**Camili**	449		712	22		35
**Otluk**	202		139	9		7
**Tepecikoren**	112		111	5		5
**Koyunevi**	90		53	4		3
**Total 2012**	3,524		4,207	207		174
**2013**	**Female**	**Engorged**	**Male**	**Female**	**Engorged**	**Male**
**Damyeri**	361	103	473	19	103	24
**Zerdali**	59	10	261	29	10	13
**Camili**	84	42	1017	46	42	48
**Otluk**	46	2	26	3	2	3
**Kizillar**	241	21	116	11	21	6
**Malihidirli**	20	-	704	1	-	35
**Total 2013**	811	178	2582	109	178	129

### Sandfly rates of infection

The TORV specific rt-RT-PCR confirmed that pools #213 and #292 were positive (Ct values < 26). Pool #10 (20 females collected in Damyeri in 2013) was also positive for TORV. Four-fold dilutions of the RNA were positive until the dilution 1:256 for the pools #213, #292, and #10.

The ZERV specific rt-RT-PCR confirmed pool #37 was positive (Ct value = 26.33), and detected ZERV RNA in 3 additional pools (#128–20 females, #342–20 males, and #374–29 females). Four-fold dilutions of the pool #37 RNA on the one hand and of pools #128, #342 and #374 on the other were positive until dilutions 1:4,096 and 1:1,1024, respectively. Pools#128, #342 and #374 consisted of sandflies trapped in 2012 in the respective villages of Damyeri, Camili and Koyunevi (Fig 1).

The rate of infection for TORV was 0.026%, for ZERV 0.035%, and for both TORV and ZERV 0.062% assuming that only one sandfly was infected in each pool.

### Virus isolation

Vero cells inoculated with pool #292 showed a clear CPE at day 6 post-inoculation (pi). Pool #213-inoculated Vero cells did not produce CPE during 4 serial passages. However, virus replication was demonstrated by RT-PCR (N-phlebo1 system, 24) starting from passage 3. CPE appeared at day 4 pi at passages 4 and 5 and virus replication was confirmed by RT-PCR. In a similar manner, pool #37- inoculated Vero cells provided a clear CPE at day 4 pi of passage 3 (RT-PCR was positive at passage 2). Neither virus isolation nor positive RT-PCR was obtained after 5 serial passages for pools #10, #128, #342, and #374.

Freeze-dried suspensions of ZERV-strain #37 (passage 8), TORV-strain #292 (passage 5) and TORV-strain #213 (passage 8) have been included in the collection of the European Virus Archive (www.european-virus-archive.com/) where they are publicly available for academic research at non-profit costs.

### Complete genome sequencing

The complete genomes of both strains (#213 and #292) of the TORV consisted of 6,456 nts, 4,326 nts and 1,702 nts for the L, M and S segment, respectively (Genbank acc. no of the strain 213; KP966619, KP966620, andKP966621; Genbank acc. no of the strain 292;KP966622, KP966623, and KP966624). The polymerase gene encoded a 6,270-nt long ORF (2,090 AA), whereas the glycoprotein gene encoded a 4,077-nt long ORF (1,359AA). The small segment encoded a 738-nt and a 780-nt long ORF which when translated corresponded to the nucleocapsid protein (246 AA) and a non-structural protein (260 AA), respectively.

The complete genome of the ZERV (strain #37) consisted of 6,403 nts, 4,202 nts and 1,907nts for the L, M and S segment, respectively (Genbank acc. KP966616, KP966617, and KP966618). The polymerase gene encoded a 6,285-nt long ORF (2,095 AA), whereas the glycoprotein gene encoded a 4,002-nt long ORFs (1,334). The small segment encoded a 942-nt and a 762-nt long ORF which were translated to a nucleocapsid protein (314AA) and a non-structural protein (254AA), respectively.

The complete genome of the CFUV consisted of 6,453nts, 4,329nts, and 1,704nts for the L, M and S segment, respectively (Genbank acc. no KR106177, KR106178, and KR106179). The polymerase gene encoded a 6270-nt long ORF (2,090AA), whereas the glycoprotein gene encoded a 4,077-nt long ORFs (1,359 AA). The small segment encoded a 738-nt and a 780-nt long ORF which were translated to a nucleocapsid protein (246AA) and a non-structural protein (260AA), respectively.

### Genetic distances

Pairwise distances of the nt- and AA- sequences are presented in S1 Table. The alignment of each gene is also available in S2 Table.

AA distances between TORV and SFSV-like viruses (SFSV, SFTV, SFCV, CFUV) were ≤25.2% (N), ≤37.8% (NS), ≤43.3% (M), ≤40.7% (Gn), ≤33.7% (Gc) and ≤20.6% (L), whereas AA distances between TORV and other phleboviruses were much higher: ≥ 42.9% (N), ≥71.4% (NS), ≥58.7% (M), ≥53.3% (Gn), ≥ 47.4% (Gc) and ≥43.9% (L).

AA pairwise distances between ZERV and viruses of the SFNV species (TOSV, THEV, SFNV, PUNV, MASV, GRAV) were ≤17.3% (N), ≤58.0% (NS), ≤42.7% (M), ≤41.8% (Gn), ≤29.5% (Gc) and ≤17.4% (L), whereas AA distances between ZERV and other phleboviruses were much higher: ≥ 40.6% (N), ≥80.9% (NS), ≥66.3% (M), ≥64.8% (Gn), ≥55.0% (Gc) and ≥44.5% (L).

Gene by gene comparative analysis showed that distances observed between ZERV and viruses belonging to the SFNV species were consistently lower than the lowest distances observed between ZERV and non SFNV-phleboviruses. The same relationship was observed with distances between TORV and SFSV-like viruses on the one hand, and TORV and non-SFSV-like viruses on the other.

These findings are supportive for (i) the inclusion of TORV in the SFSV species complex (SFSV, SFTV, SFCV, CFUV), (ii) the inclusion of ZERV in the SFNV species complex (TOSV, THEV, SFNV, PUNV, MASV, GRAV).

### Phylogenetic analysis

**Fig 2:**
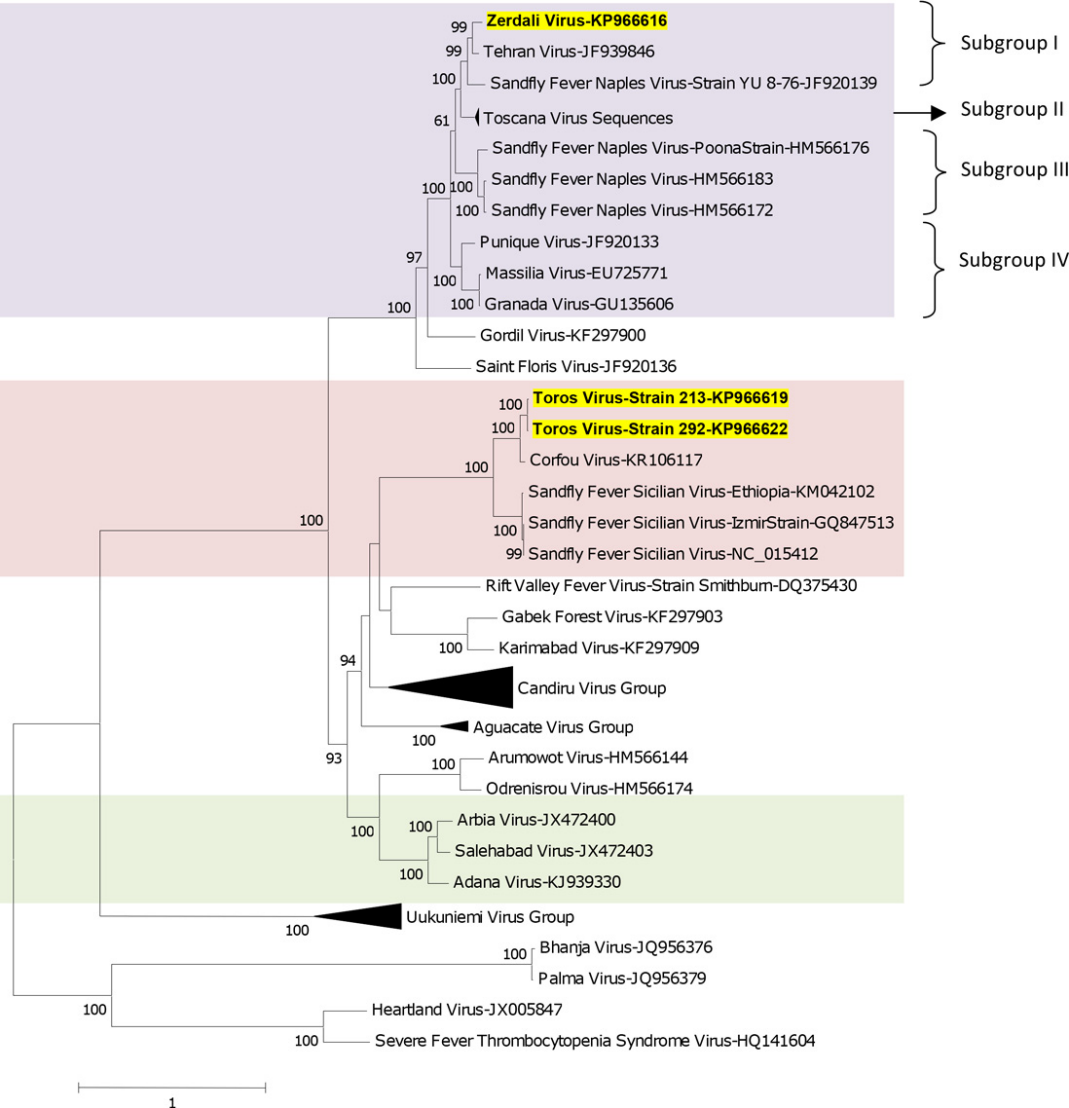
The Maximum likelihood phylogenetic analysis of the phlebovirus polymerase sequences. The Genbank accession numbers of all the phleboviruses included in the analysis can be found in S2 Table.

**Fig 3:**
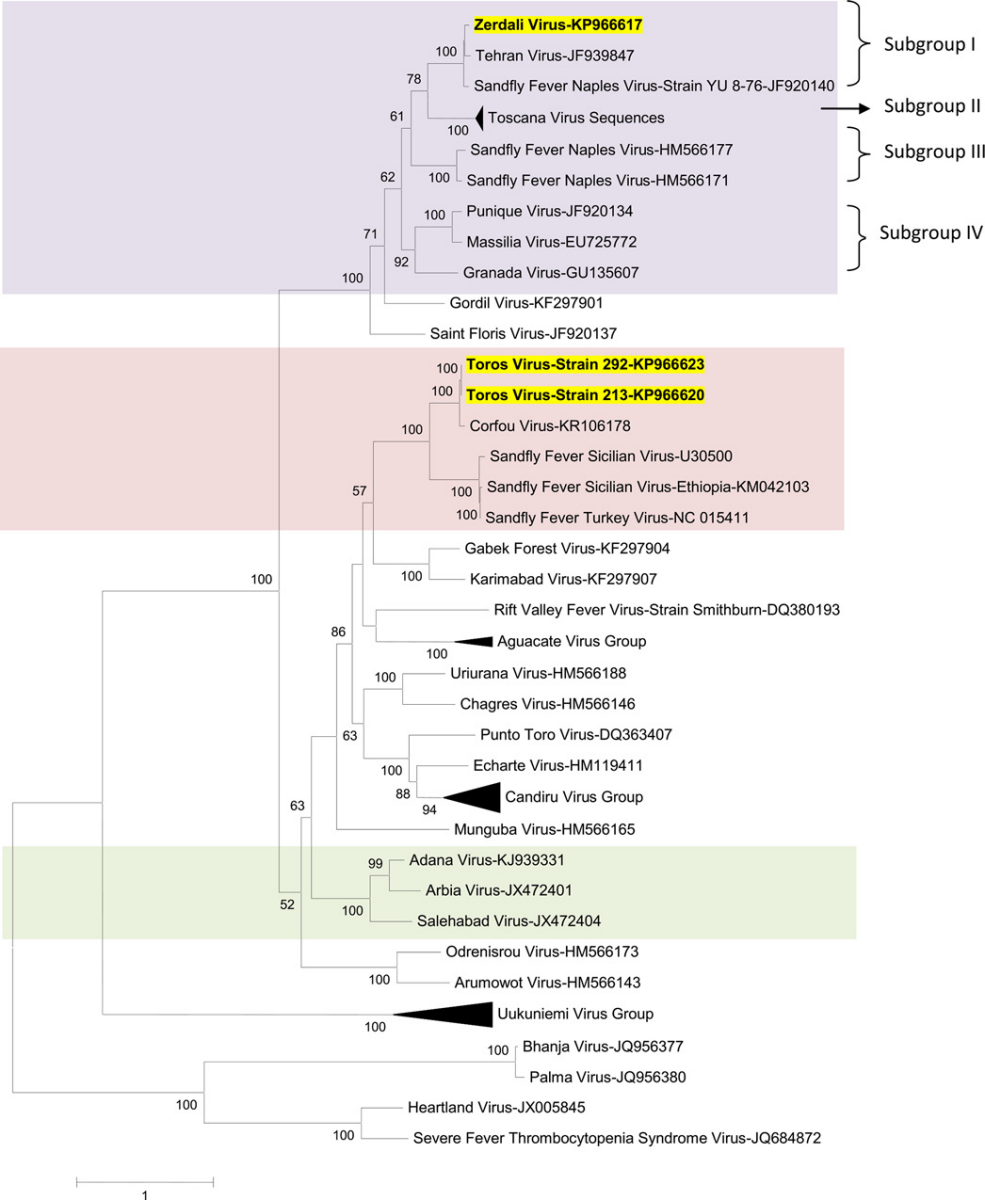
The Maximum likelihood phylogenetic analysis of the phlebovirus glycoprotein n (Gn) sequences. The Genbank accession numbers of all the phleboviruses included in the analysis can be found in S2 Table.

**Fig 4:**
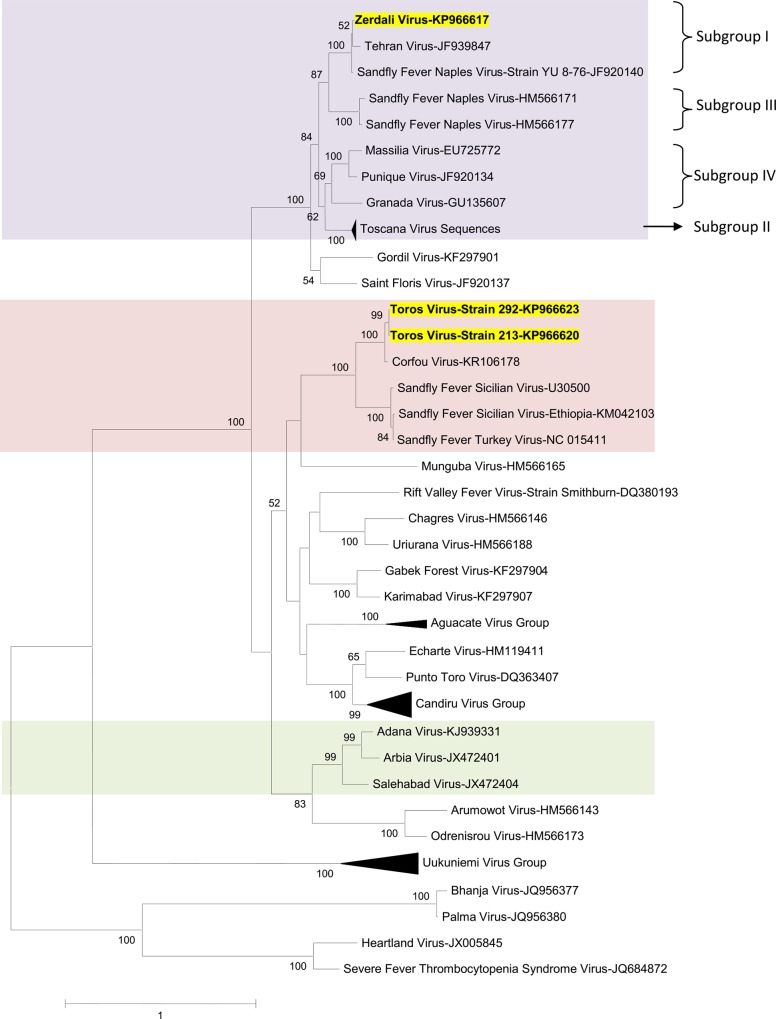
The Maximum likelihood phylogenetic analysis of the phlebovirus glycoprotein c (Gc) sequences. The Genbank accession numbers of all the phleboviruses included in the analysis can be found in S2 Table.

**Fig 5:**
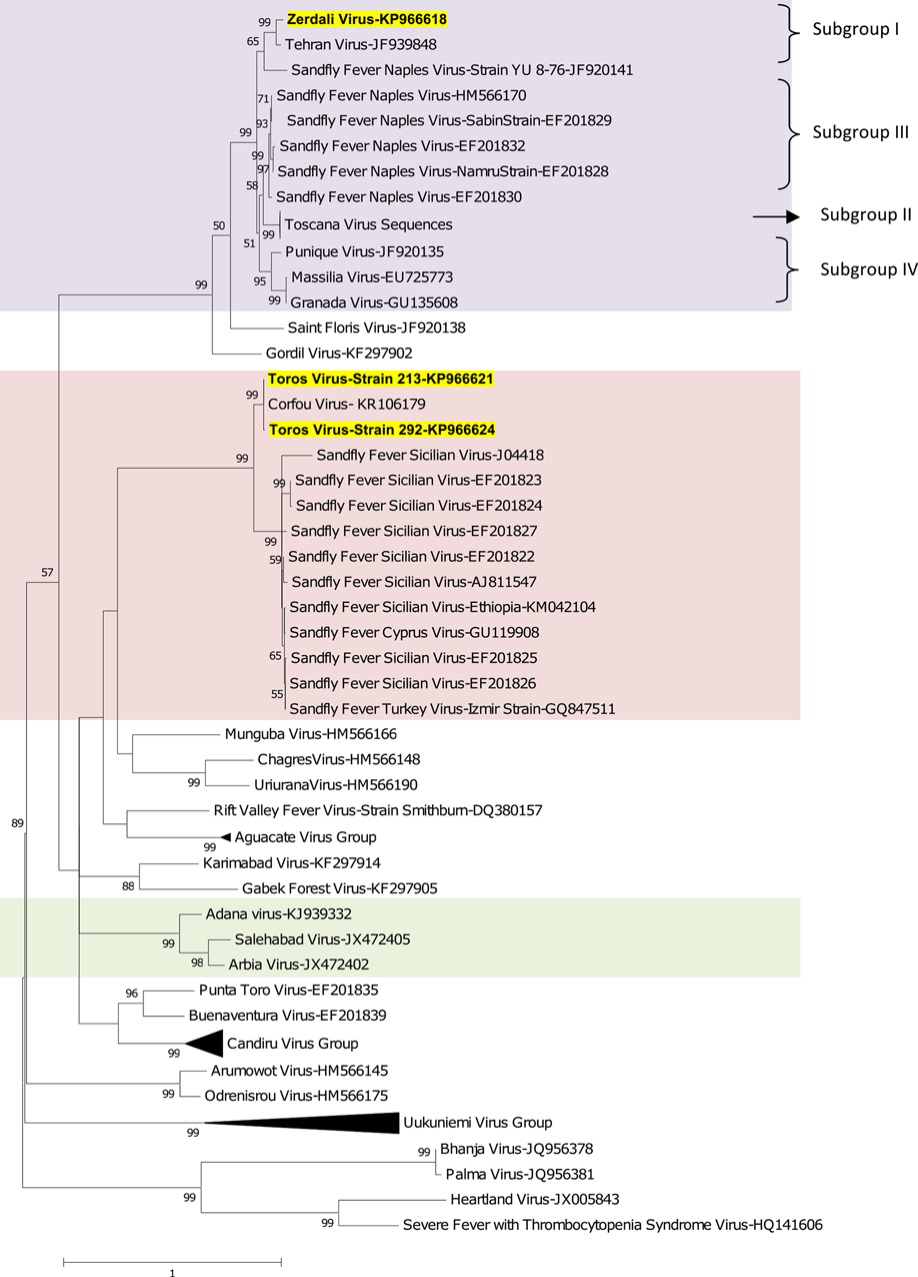
The Maximum likelihood phylogenetic analysis of the phlebovirus nucleocapsid protein sequences. The Genbank accession numbers of all the phleboviruses included in the analysis can be found in S2 Table.

**Fig 6:**
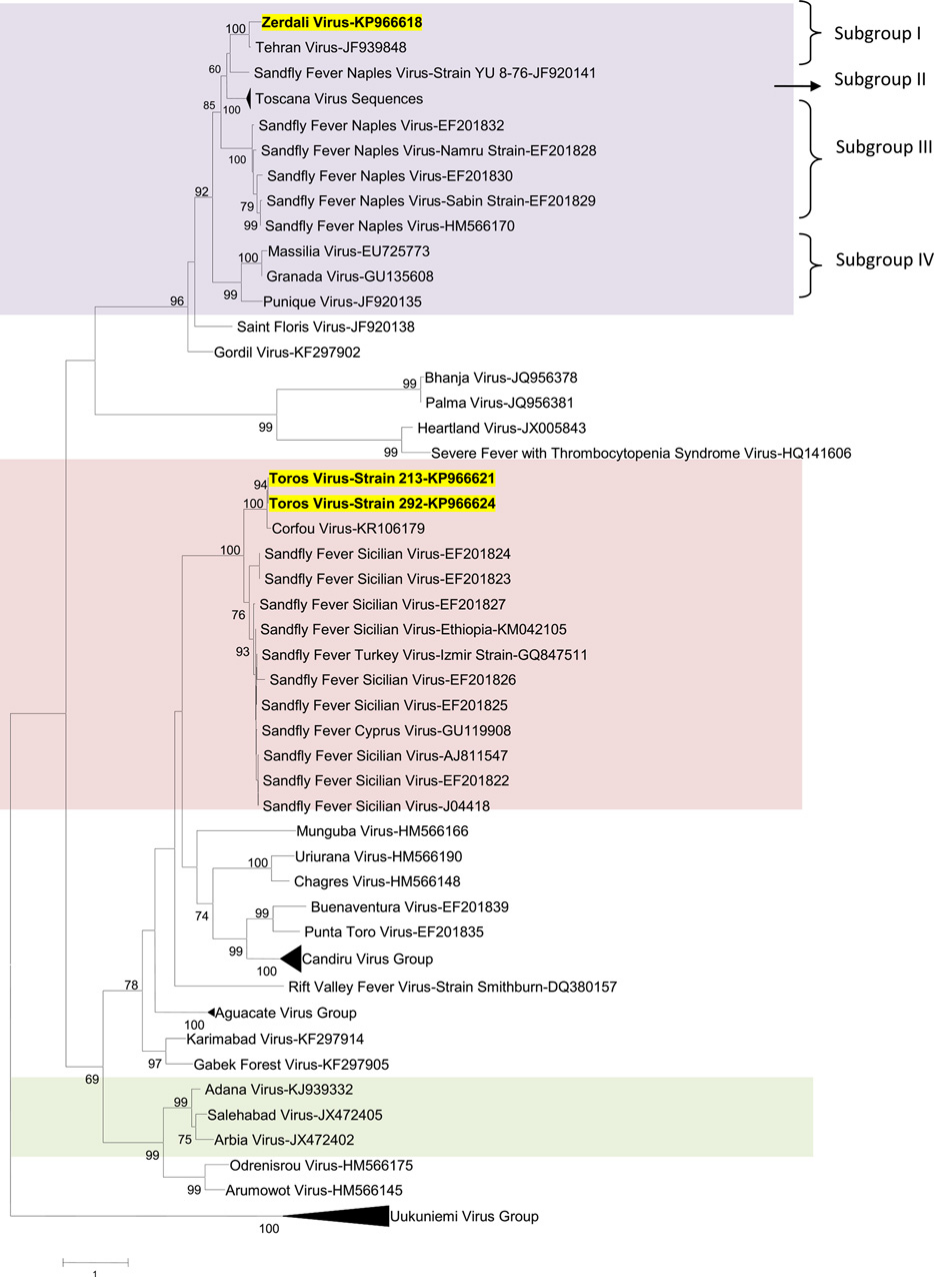
The Maximum likelihood phylogenetic analysis of the phlebovirus non-structural protein sequences. The Genbank accession numbers of all the phleboviruses included in the analysis can be found in S2 Table.

Regardless of the gene used for phylogenetic analysis, and the tree-building programme (i.e. NJ or ML) TORV clustered with SFSV, CFUV and the other SFS-like viruses (from Turkey, Cyprus, Ethiopia) with bootstrap values ≥ 99% (Figs 2, 3, 4, 5 and 6). TORV consistently grouped together with CFUV (≥ 99%bootstrap) forming a subgroup within the SFS-like viruses that is distinct from the second subgroup including SFSV and related genotypes originating from Italy, Turkey, Cyprus, and Ethiopia. The stability of the topology and relationships between TORV and most closely related viruses suggested that the TORV genome did not contain evidence of genetic recombination or reassortment. Likewise, no recombination events were detected using any of the 6 algorithms implemented in RDP4.

ZERV consistently grouped together with THEV and Naples virus strain Yu_8–76 (subgroup I), with bootstrap values at ≥ 99 for L, Gn and Gc, and with lower values for N and Ns. Within this species, there were 3 other subgroups: (i) subgroup II: Toscana viruses; (ii) subgroup III: Naples viruses (except for Yu_8–76 included in subgroup I); (iii): subgroup IV: Granada, Massilia and Punique viruses.

Partial sequences were obtained in the polymerase gene for pool #128 (505 nt) and #10 (379 nt), and in the nucleoprotein for pools #128 (280 nt), #342 (438 nt), and #374 (245 nt). Partial polymerase sequences of the pools of #128 and #10 were identical (except 2 nts and 1 nt, respectively but 100% identical in AA) with ZERV and TORV, respectively. The partial nucleoprotein sequence from the pool #128 was also identical with the ZERV (except 1 nt but 100% identical in AA). However, the partial nucleoprotein sequences from the pool #342 (6 nt and 3 AA different) and #374 (49 nt and 2 AA different) were not identical with ZERV although originating from neighbouring localities (Fig 1); this suggests that there may be topotypes that remain to be identified and characterised.

### Genotyping the sandflies of virus-positive pools

Genotyping was performed for 7 pools. The species composition of the pools and number of reads are shown in Table 2. NGS reads were compared with available sequences in Genbank (Genbank accession numbers: KT634318, KF483675, KR349298, JQ769142, KF137560, KJ481126) by Blastn using the CLC Genomic Workbench 6.5. The species were determined when the consensus sequences had ≥98% similarity with the regional reference sequences except *Sergentomyia sp*. sequences which had ≥85% similarity with *S*. *dentata* from Adana (Genbank accession numbers: KU659595, KU659596, KU659597, KU659598; release date 01 July 2016). Therefore we indicated these sequences as *Sergentomyia sp*.

**Table 2:** Genotyping of sandflies in the virus positive pools.

		Number of reads			
Pool	Region	*P*. *tobbi*	*P*. *perfiliewi* s. l.	*P*. *papatasi*	*Sergentomyia* sp.
213	Damyeri	486	201	-	-
292	Damyeri	5	15	12	-
10	Damyeri	13	357	42	-
37	Zerdali	3153	24	159	65
128	Damyeri	650	952	327	28
342	Camili	63	299	-	-
374	Koyunevi	282	-	201	-

## Discussion

Although there are published serological data [13] and a recent report of detection of TOSV [19] in the Adana, Mediterranean region of Turkey there have been no previous reports of virus isolation. To further understand the presence of sandfly-borne phleboviruses that circulate in this endemic region for leishmaniasis [31], close to the border of Syria where many refugee camps are settled, we organised field-study campaigns in 2012 and 2013. The TORV and ZERV that were isolated during our study were most closely related to but distinct from SFS- and SFNV- like viruses, respectively. Studies conducted in 2012 led to the isolation of ADAV a novel putative member of the *Salehabad* species [20]. These results demonstrate that 3 phleboviruses belonging to 3 different genetic lineages co-circulate in the population of sandflies in this geographic area. Cumulative data resulting from this study and that of [20] enabled estimation of infection rates in sandflies (0.07% for sandfly-borne viruses in this region of Turkey) which is in the same order of magnitude as previously reported in France, Tunisia, Spain and Italy [2, 23, 32,33, 34].

Regardless of the gene used for analysis TORV and CFUV were grouped together in a sublineage that is clearly distinct from that including all other SFS-like viruses. CFUV was isolated from *Phlebotomus major* sensu lato [35] trapped in the eponymous Greek island. Interestingly, TORV has been isolated from two pools that contained *P*. *perfiliewi* sensu lato and *P*. *tobbi*, both belonging to the *Larroussius* subgenus as *P*. *major* sensu lato. Similarly, SFTV was detected in *P*. *major* sensu lato [21]. In contrast, other SFSV strains were isolated from *P*. *papatasi* that belongs to the *Phebotomus* subgenus which is distinct from the *Larroussius* subgenus [36]. Therefore, the two subgroups of viruses might reflect vector properties, with CFUV/ TORV associated with the *Larroussius* subgenus whereas other SFSV viruses are associated with *P*. *papatasi* with the exception of SFTV association with *P*. *major* sensu lato. Vector-virus association needs to be studied in a more detailed manner in order (i) to determine unambiguously the sandfly species transmitting these newly described viruses, (ii) and to verify our hypothesis that virus subgroups within viral species might be linked to specific vectors belonging to distinct taxonomic entities.

Additional field studies combined with experimental studies using sandfly colonies need to be initiated to understand the parameters driving vector capacity and competence for different strains of viruses.

ZERV was consistently grouped together with THEV and Naples virus strain YU-8-76. Interestingly, THEV and the Serbian isolate Yu 8/76 apparently do not require expression of the NSs ORF, since their replication is not impaired by the presence of either an early stop codon or a large truncation [37], whereas there is no such impairment or truncation in the ZERV genome which has a complete NSs ORF. Similar observations were also reported for a naturally attenuated RVFV strain (clone 13) that has a large in-frame deletion in the NSs coding region [37, 38].

Within the SFNV species, it is possible to discriminate 4 sublineages (I to IV); we propose to assign ZERV to sublineage I, where it was most closely related with THEV (Figs 2, 3, 4, 5 and 6). THEV was isolated from *P*. *papatasi* sandflies in Iran in 1959 [39], whereas YU-8-76 strain of SFNV was isolated from *P*. *perfiliewi* sensu lato trapped in Serbia in 1976 [37]. Subgroup III appears to be associated with *P*. *papatasi*, whereas subgroups II and IV appear to be associated with vectors belonging to the subgenus *Larroussius*. Subgroup I may be associated with *Larroussius* except THEV isolated in *P*. *papatasi*. Only *P*. *tobbi* was found to be present in all of the four ZERV positive pools. The same comment formulated above concerning the need for experimental studies to understand species-related competence and specificity of sandflies applies here.

Genetic and phylogenetic analyses support the fact that both ZERV and TORV should be considered as new strains within pre-existing SFNV species and the yet to be recognised species including SFSV and CFUV, respectively.

To date, SFSV and CFUV are listed as tentative species by the ICTV [1]. This study, based on complete genome sequences, suggests that all these viruses should be considered as members of the same species which could be further subdivided into CFUV / TORV, and SFSV / SFS-like viruses. A written proposal will be submitted to the *Bunyaviridae* Study Group of the ICTV.

During this two-year study, 48% of the sandflies were trapped from Damyeri village where the ecological conditions were no different from those observed in other sampling stations. However, in Damyeri, the number of domestic animals (sheep, goats, and cows) was much higher than in other localities and these animals were constantly in the close vicinity of houses producing droppings which are known to be favoured breeding sites for sandflies or we set the traps very close to the possible breeding sites by chance therefore we got higher numbers of sand flies for this village. Thus, human exposure to sandflies might be greater in Damyeri than other sampling stations.

Whether TORV and ZERV can infect humans and cause diseases such as sandfly fever is currently unknown and remains to be investigated. After the outbreak of sandfly fever occurred in Adana in 2008, specific IgM against SFSV and/or SFCV was detected in acute cases by mosaic-immunofluorescence test, although the cause of the epidemic was not formally established through virus isolation or molecular detection with sequence confirmation [13]. The region where this outbreak occurred is located less than 25 km away from our trapping stations. Despite the fact that this study does not provide results supporting that both newly discovered viruses are human or animal pathogens, both TORV and ZERV belong to genetic groups that include several human pathogens. There is no doubt that SFSV "historic strains" were causing massive outbreak of debilitation and incapacitating disease [12, 40, 41]. Moreover, SFCV was also isolated from human cases [42, 43], as well as SFTV [13]. Antibodies against CFUV / SFSV were reported in humans living in mainland Greece and on Corfu Island using the immunofluorescence assay (IFA) [44]. Viral RNA of Chios virus, closely related to CFUV, was detected in the CSF of a patient presenting with severe encephalitis (Papa and Pavlidou, 2003, Genbank no, AY293623). In contrast, specific antibodies were never described in humans for THEV [12], although SFNV a close relative of THEV and subsequently ZERV was undoubtedly the cause of explosive outbreaks in newcomers to endemic areas during summertime [9]. Importantly, although serological studies have not yet been reported we do have serological data to support the concept that the newly isolated ZERV and TORV can infect vertebrates. However, whether or not these vertebrates do play a reservoir or amplifying role is not yet clear. Interestingly, although TORV and ZERV genomic RNA was detected in female pools of sandflies, both viruses were only isolated from male pools. Based on current knowledge it is not known how male sandflies become infected. Whilst, transovarial transmission seems a likely possibility it is not yet known how significant or efficient this mechanism of transmission is in natural habitats. However, laboratory experiments have shown that the rates of infection amongst offspring are low and show a decline from the first generation to ongoing generations. Other studies suggest that venereal (horizontal) transmission from infected males to uninfected females by mating and transstadial transmission of TOSV in diapausing *Phlebotomus perniciosus* larvae [9] may also contribute to long-term virus survival. From what is currently known and in the absence of defined vertebrate reservoirs, maintenance and transmission of sandfly-borne phleboviruses appears to depend on the abundance and accessibility of appropriate vector species. This lack of available knowledge of virus transmission and the virus maintenance mechanisms clearly need to be investigated both in natural habitats and under experimental conditions. Future studies are planned to examine female sandfly salivary glands and heads to look for the presence of infectious virus.

Recent studies have detected sequences compatible with the existence of many putative new phleboviruses transmitted by sandflies in the Old World. In most of the cases, they were only partial sequences in the polymerase or the nucleoprotein genes. To date these limited genetic data, preclude classification by ICTV. This situation applies for viruses that may belong to (i) the SFNV species such as FERV [2], Provincia virus [48], Girne1 virus [18], and Saddaguia virus [45], (ii) the SALV species such as Adria virus [46, 47] Olbia virus [48] and Edirne virus [18], and to (iii) the SFSV / CFUV complex such as Chios virus (Papa and Pavlidou, 2003, Genbank no, AY293623), Utique virus [4], Girne2 virus [18], SFS-like viruses [4, 42, 43, 49]. Accordingly, although our knowledge of sandfly-borne phleboviruses is more extensive than it was a half-decade ago; efforts to isolate virus strains and determine their complete sequence should continue. Since virus taxonomy for the *Phlebovirus* genus still relies on neutralisation-based antigenic relationships, virus isolation is also essential. Nevertheless, the criteria for taxonomy appear to be evolving towards full-length genome comparative analysis.

In conclusion, the results obtained in this study together with previously published results [20] demonstrate that (i) at least 3 different phleboviruses are co-circulating in phlebotomine sandfly populationsin the Adana region of Mediterranean Turkey; (ii) these new viruses belong to 3 distinct but closely related phylogenetic groups or species (SFNV, SALV, SFSV / CFUV); (iii) all the closely related viruses are known to be sandfly-borne arboviruses; (v) we have evidence of vertebrate seroprevalence for this group of viruses. Thus whilst it is indirect, the evidence for TORV and ZERV being arboviruses is compelling. It is also important to emphasise that although >10,000 sandflies were tested in this study; TOSV was neither detected nor isolated from the field samples. This challenges recent reports of TOSV-specific antibodies in blood donors from this region of Turkey [17], and TOSV RNA / TOSV IgG in dogs from Mersin and Adana [19]. However, we recognise that the sampling points in these earlier studies do not overlap with those selected for our investigations.

## Supporting Information

S1 Table Amino acid and nucleotide pairwise distances between phleboviruses including the Zerdali virus and Toros virus. (XLSX)

S2 Table The alignments of L, Gn, Gc, N, and Ns genes of the phleboviruses including the Zerdali virus and Toros virus. (MAS)
